# Variation in plasma 25-hydroxyvitamin D2 and D3 in normal pregnancy with gestational age, sampling season, and complications: A longitudinal cohort study

**DOI:** 10.1371/journal.pone.0231657

**Published:** 2020-04-17

**Authors:** Astrid Bakke Orvik, Malene Rohr Andersen, Palle Skov Bratholm, Katrine Kaare Hedengran, Christian Ritz, Steen Stender, Pal Bela Szecsi

**Affiliations:** 1 Department of Gynecology and Obstetrics, Copenhagen University Hospital Holbæk, Holbæk, Denmark; 2 Department of Clinical Biochemistry, Herlev and Gentofte Hospital, Hellerup, Denmark; 3 Department of Clinical Biochemistry, Copenhagen University Hospital Holbæk, Holbæk, Denmark; 4 Department of Gynecology and Obstetrics, Copenhagen University Hospital Nordsjælland, Hillerød, Denmark; 5 Department of Nutrition, Exercise and Sports, University of Copenhagen, Copenhagen, Denmark; University of Queensland, AUSTRALIA

## Abstract

**Introduction:**

Low levels of vitamin D in pregnancy have been associated with the risk of a variety of pregnancy outcomes. Few studies have investigated vitamin D concentrations throughout pregnancy in healthy women, and most guidelines recommend high vitamin D levels. In the present study, we investigated 25-hydroxyvitamin D concentrations in healthy Caucasian Danish women in relation to season, gestational age and possible vitamin D-linked complications.

**Materials and methods:**

Eight hundred and one healthy Caucasian Danish women with an expected normal pregnancy were recruited among 2147 women attending first trimester screening. Seven blood samplings were planned throughout the pregnancy and delivery period. The 25-hydroxyvitamin D2 (25(OH)D2) and 25-hydroxyvitamin D3 (25(OH)D3) concentrations were measured by LC-MS/MS and total 25-hydroxyvitamin D (25(OH)D) were calculated.

**Results:**

A total of 3304 samples from 694 women were available for 25(OH)D measurements. The mean (25^th^-75^th^ percentiles) concentrations of 25(OH)D, 25(OH)D3, and 25(OH)D2 were 54.6 (38.8–68.6) nmol/L, 52.2 (36.4–66.4) nmol/L, and 2.4 (2.2–2.2) nmol/L, respectively. Season was the strongest predictor of 25(OH)D concentration, with the lowest values observed in winter and spring, where only 42% and 41% of samples, respectively, were above 50 nmol/L. Nearly all women had values below the suggested optimal level of 75 nmol/L, independent of season. 25(OH)D peaked at gestational weeks 21–34. Plasma 25(OH)D2 levels were low in all seasons. Women with complications during pregnancy had higher 25(OH)D (estimated difference 9.8 nmol/L, standard error 2.7, p<0.001) than did women without complications, and women giving birth vaginally had lower 25(OH)D than did those delivering via elective (10.0 nmol/L, standard error 2.1, p<0.001) or emergency cesarean section (6.8 nmol/L, standard error 2.2, p<0.001).

**Conclusion:**

The 25(OH)D concentrations vary with both season and gestational age. Healthy women had lower 25(OH)D concentrations than recommended, without an association with an increased risk of pregnancy complications. Guidelines for vitamin D in pregnancy may require revision.

## Introduction

During the past 15 years, there has been much focus on the possible effects of vitamin D on a wide range of diseases and conditions [1–4]. Vitamin D is available in two distinct forms, vitamin D2 (ergocalciferol) and D3 (cholecalciferol). Vitamin D3 is both indigested, but is primarily produced in the epidermis from 7-dehydrocholesterol by sufficient sunlight irradiation (the ultraviolet B component). Vitamin D2 is only indigested from irradiated plant sterols (ergosterol). Both forms are subsequently hydroxylated in the liver and other tissues to 25-hydroxyvitamin D3 (25(OH)D3) and 25-hydroxyvitamin D2 (25(OH)D2). The main 25(OH)D forms in circulation are bound to vitamin D-binding protein, which enables a relatively long half-life (2–3 weeks), and this form has therefore been considered the main marker for vitamin D status [5]. The biologically active metabolite, 1,25-dihydroxyvitamin D, has a much shorter half-life (1–2 days). At northern latitudes, insufficient solar UVB radiation from October through March diminishes the cutaneous synthesis of vitamin D3, normally accounting for 95% of vitamin D [6]. The remaining 5% is acquired through the diet. In Denmark, in contrast to many other countries, still few food products are fortified [7]. At the sampling period for this study, only a handful of margarine products had obtained permission for fortification and the maximal concentration was 10 μg per 100 g (personal communication, Danish Veterinary and Food Administration). In pregnancy, the need for both calcium and vitamin D is increased due to fetal development, and vitamin D receptors are expressed in the placenta [8, 9]. Low levels of vitamin D during pregnancy have been reported globally [10]. Several explanations has been proposed, including changes in plasma composition, decreased exposure to sun, and rise in vitamin D-binding protein levels [5, 9]. Low levels have been associated with the risk of a variety of conditions and pregnancy outcomes, e.g., preeclampsia, gestational diabetes, preterm birth, cesarean section, low birth weight, asthma or recurrent wheezing, and impaired neurological development in children [2, 6, 11, 12]. Few studies have investigated vitamin D concentrations throughout pregnancy in healthy women. Most studies have used immunological methods to measure 25(OH)D, which have greater uncertainty, do not differentiate between 25(OH)D2 and 25(OH)D3, and yield method-dependent results [13]. The gold standard is liquid chromatography tandem mass spectrometry (LC-MS/MS) [13]. Since recommendations on supplement usage, diet, and sun exposure vary from country to country, findings are not necessarily generalizable. In the present study, we investigated 25(OH)D, 25(OH)D2, and 25(OH)D3 concentrations in healthy Caucasian Danish women in relation to season, gestational age, and possible vitamin D-linked complications.

## Materials and methods

### Study participants and samples

**Fig 1:**
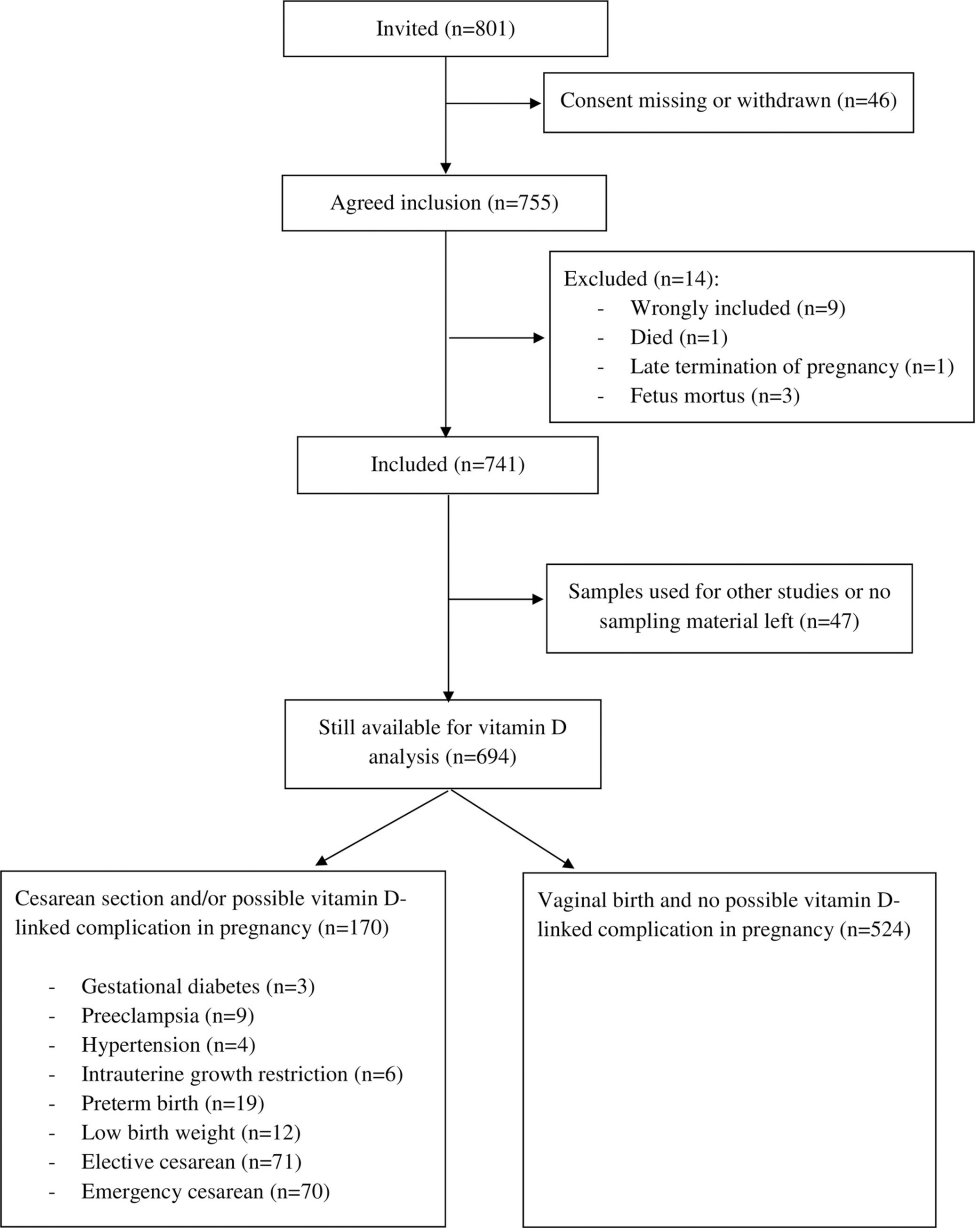
Consort flow chart.

A total of 801 healthy Caucasian women above 18 years of age, with an expected normal singleton pregnancy, were recruited among 2147 women attending first trimester screening between June 2006 and October 2007, in Copenhagen at latitude 56° N, as previously described in detail [14]. A total of 755 women agreed to be included, and 14 were excluded. Samples from 694 women were still available for 25(OH)D measurements (Fig 1). All women invited to participate had singleton pregnancies with normal nuchal translucency scans, as well as normal free β-human chorionic gonadotropin and pregnancy-associated plasma protein. All clinical data were obtained from pregnancy charts and medical records. Complications occurring during pregnancy, potentially linked to low vitamin D levels, were defined as gestational diabetes, preeclampsia, hypertension (blood pressure above 140/90 mmHg), intrauterine growth restriction, preterm birth (< gestational week 37+0), low birth weight (<2500 g regardless of gestational age), and elective or acute cesarean section. Respondents were classified into five social groups based on employment grade, job title, and education: I) executive managers and/or academics; II) middle managers and/or those with 3–4 years of further education; III) other white-collar workers; IV) skilled blue-collar workers; and V) semi-skilled or unskilled workers. The study population was similar to that of all women delivering at our hospital during the same period with respect to age, parity, and self-reported pre-pregnancy body mass index (BMI). Each woman was scheduled for seven blood samples at the following time points: gestational weeks 13–20, 21–28, 29–34, and 35–42; at active labor or cesarean section; and on the first and second day after delivery. Sampling seasons were defined as winter (December-February), spring (March-May), summer (June-August), and autumn (September-November). Blood samples and clinical data were collected between June 2006 and October 2007.

### Vitamin D analysis

Blood samples were collected and transported to the laboratory, where they were stored at -80ºC until use. Plasma 25(OH)D2 and 25(OH)D3 concentrations were measured after ethanol precipitation, liquid-liquid extraction, and LC-MS/MS with an in-house assay using deuterated 25(OH)D3 and 25(OH)D2 as internal standards and detection of the 401.2→383.2 and 413.1→355.2 mass transitions for 25(OH)D3 and 25(OH)D2, respectively. Calibrators were traceable to NIST SRM 972, and the method was DEQAS-proficiency certified. The inter-assay precision values were less than 10% for 25(OH)D3 at 27 and 85 nmol/L and less than 20% for 25(OH)D2 at 32 and 130 nmol/L. The limit of quantitation was estimated to be 9 nmol/L for 25(OH)D3 and 2.5 nmol/L for 25(OH)D2 based on 10 times the standard deviation of the area of the chromatogram at baseline. The 25(OH)D concentrations were calculated by adding the results of 25(OH)D2 and 25(OH)D3. The results below the limit of quantitation were set to 10% lower than this limit. The local reference interval for 25(OH)D2 + 25(OH)D3 was 50–160 nmol/L. The results were not made available to the clinicians during the study.

### Statistical calculations

Data were analyzed using SPSS Version 25 (IBM, Armonk, NY, USA) and R [15]. Differences in mean 25(OH)D levels between groups and associations with predictors were estimated using a linear mixed model, which was fitted using restricted maximum likelihood estimation. The model included all predictors simultaneously with the only exception when a continuous predictor was replaced by its dichotomized version (for age and BMI), ensuring full adjustment for measured confounding. In total three linear mixed models were fitted. The following predictors (fixed effects) were simultaneously investigated: gestational period; season; possible vitamin D-linked complications in pregnancy; mode of delivery; mode of conception; smoking; BMI or BMI group; parity; age or age group; and social class. Participant-specific random intercept effects were also included in the linear mixed model to capture the biological between-participant variation. Estimated mean differences and slope coefficients were reported. Based on the linear mixed models, post hoc pairwise comparisons using approximate t-tests were performed to quantify relevant differences. A significance level of 0.05 was used to declare a significant result. P-values were not multiplicity adjusted due to the exploratory and hypothesis-generating nature of the study. Reference intervals (2.5^th^ and 97.5^th^ percentiles) were calculated for gestational period and sampling season for women with vaginal birth and without possible vitamin D-linked pregnancy complication by the non-parametric bootstrap method with 500 iterations in REFVAL software, version 4.11, according to the recommendations of the International Federation of Clinical Chemistry. Outliers were removed using Horn’s algorithm (fence factor 1.5). If fewer than 40 cases are present, only a descriptive 95 inter-percentile range is given.

### Ethical approval

The study was conducted in accordance with the Declaration of Helsinki. The participants provided written informed consent, and the study was approved by the Local Research Ethics Committee (Den Videnskabsetiske Komité for Københavns Amt No. KA 05065) and the Danish Data Protection Agency.

## Results

In total, 3304 samples from 694 of the 801 originally enrolled women were available for 25(OH)D measurement. Of these, 2403 were samples from 524 women who delivered vaginally and without possible vitamin D-linked complications. 170 women (901 samples) had cesarean section and/or possible vitamin D-linked complications (Fig 1). Each woman contributed an average of 4.8 (range 1–7) samples. The cohort had a mean (standard deviation) age of 32 (4.3) years, BMI of 22 (3.1) kg/m^2^, gestational weight of 3575 (477) grams, and gestational age of 281 (9) days. Demographics in relation to 25(OH)D concentrations are shown in Table 1. The mean (25^th^-75^th^ percentiles) concentrations of 25(OH)D, 25(OH)D3, and 25(OH)D2 in the cohort were 54.6 (38.8–68.6) nmol/L, 52.2 (36.4–66.4) nmol/L and 2.4 (2.2–2.2) nmol/L, respectively.

**Table 1:** Characteristics of 694 healthy pregnant Caucasian women in relation to plasma 25(OH)D concentrations.

	Women		Samples		25(OH)D, nmol/L		25(OH)D, % of samples		
	n	%	n	%	mean	SD	<25	<50	<75
**Overall**	694		3304		54.6	(21.4)	7.5	44.2	83.4
Gestational week	-		3304						
<13^1^	25	-	25	0.8	54.4	(17.0)	0.0	44.0	92.0
13–20	452	-	622	18.8	60.1	(18.8)	2.6	28.0	81.5
21–28	478	-	630	19.1	60.0	(21.8)	3.8	33.8	76.2
29–34	289	-	293	8.9	58.6	(21.2)	4.1	36.5	80.9
35–42	382	-	405	12.3	53.7	(21.0)	6.9	47.7	82.7
Delivery	488	-	488	14.8	52.3	(22.5)	10.9	52.3	83.6
1^st^ day after delivery	490	-	490	14.8	46.4	(19.8)	13.9	60.6	91.2
2^nd^ day after delivery	350	-	351	10.6	47.2	(19.8)	13.4	59.5	90.3
**Sampling season**	-		3304						
Winter	493	-	934	28.3	47.6	(19.3)	11.9	58.1	91.3
Spring	407	-	893	27.0	47.4	(19.6)	12.0	59.0	90.8
Summer	346	-	633	19.2	68.8	(20.2)	0.9	16.7	64.9
Autumn	483	-	844	25.5	59.1	(19.6)	2.8	33.5	80.5
**Possible vitamin D-linked complication**	694		3304						
Not present	652	93.9	3105	94.0	54.2	(21.3)	7.9	44.5	83.8
Present	42	6.1	199	6.0	59.8	(21.7)	1.5	38.7	76.9
**Mode of delivery**	694		3304						
Vaginal birth	553	79.7	2550	77.2	54.0	(21.1)	7.5	45.1	83.9
Elective cesarean	71	10.2	409	12.4	55.1	(23.3)	9.3	42.8	82.6
Emergency cesarean	70	10.1	345	10.4	57.8	(20.9)	5.8	38.6	80.3
**Mode of conception**	694		3304						
Spontaneous	677	97.6	3217	97.4	54.5	(21.4)	7.5	44.3	83.4
Stimulation	1	0.1	3	0.1	60.5	(12.2)	0.0	33.3	100.0
In-vitro fertilization	9	1.3	49	1.5	56.0	(19.1)	8.2	36.7	85.7
Insemination	7	1.0	35	1.1	55.5	(21.6)	5.7	42.9	77.1
**Age** (years)	694		3304						
<20	1	0.2	7	0.2	74.0	(18.9)	0.0	0.0	42.9
20–29	200	28.8	967	29.3	54.5	(21.9)	8.5	45.5	83.7
30–39	472	68.0	2230	67.5	54.4	(21.2)	7.3	43.9	83.4
≥40	21	3.0	100	3.0	56.7	(20.3)	4.0	39.0	83.0
**Parity**	694		3304						
0	302	43.5	1516	45.9	56.5	(20.7)	5.8	40.1	81.9
1	280	40.3	1290	39.0	54.2	(22.1)	7.9	45.5	82.9
2+	112	16.1	498	15.1	49.7	(20.7)	11.6	53.0	88.8
**Body mass index (kg/m**^**2**^**)**	682		3251						
Underweight <18.5	45	6.6	221	6.8	55.3	(21.9)	9.0	40.7	80.1
Normal weight 18.5–24.9	510	74.8	2406	74.0	55.7	(21.6)	7.1	41.7	82.3
Overweight 25–29.9	111	16.3	539	16.6	50.7	(19.4)	7.4	52.3	89.1
Obese 30–34.9	14	2.0	71	2.2	49.0	(22.8)	14.1	59.2	84.5
Severely obese ≥35	2	0.3	14	0.4	29.9	(9.2)	42.9	100.0	100.0
**Smoking habits**	694		3304						
Non-smoker	611	88.0	2938	88.9	54.5	(21.1)	7.2	44.3	83.5
Smoker	83	12.0	366	11.1	55.0	(23.4)	9.8	42.6	82.0
**Social status**^2^	694		3304						
I	121	17.4	569	17.2	53.1	(20.9)	7.6	45.2	87.5
II	184	26.5	941	28.5	56.0	(21.4)	7.3	40.9	81.1
III	163	23.5	716	21.7	56.5	(21.0)	5.3	40.4	82.7
IV	104	15.0	501	15.2	53.5	(22.0)	10.0	47.3	81.6
V	122	17.6	577	17.5	52.1	(21.3)	8.3	50.4	85.3

The 25(OH)D concentrations in different sociodemographic subgroups of the total study population. The data are presented as the count, percentage (%), or mean and standard deviation (SD).

^1^ Not used in the mixed model calculation.

^2^ Respondents were classified into five social groups based on employment grade, job title, and education: I) executive managers and/or academics; II) middle managers and/or those with 3–4 years of further education; III) other white-collar workers; IV) skilled blue-collar workers; and V) semi-skilled or unskilled workers.

For women without any complications and vaginal birth, the mean concentrations (25^th^-75^th^ percentiles) of 25(OH)D, 25(OH)D3, and 25(OH)D2 were 53.8 (38.2–67.3) nmol/L, 51.4 (35.7–65.1) nmol/L, and 2.4 (2.2–2.2) nmol/L, respectively. For women with complications or cesarean section, the values were 56.7 (40.0–71.5) nmol/L, 54.3 (37.8–69.0) nmol/L, and 2.4 (2.2–2.2) nmol/L, respectively. The 25(OH)D2 was above the limit of quantification in 355 samples from 258 women. The concentrations were low in all seasons and gestational age periods, with a maximal value of 9.1 nmol/L.

**Fig 2:**
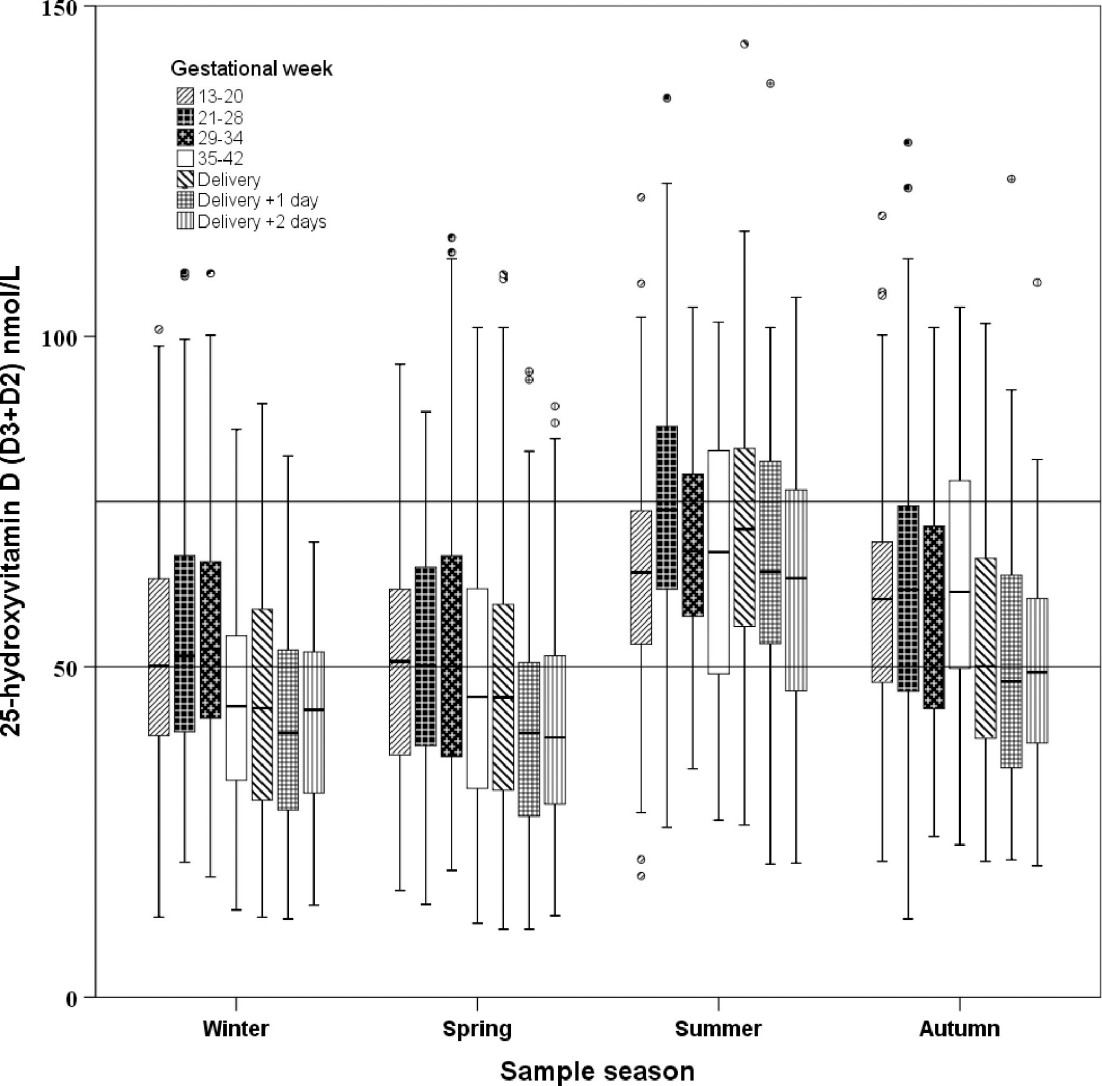
Gestational age and season-specific variation in 25(OH)D in plasma in women with vaginal birth and no possible vitamin D-linked complication in pregnancy. The 25(OH)D concentrations in 2403 plasma samples from 524 healthy, pregnant Caucasian women. The box plots represent the range of data from the 25^th^ to the 75^th^ percentile, while the bar in the middle of each box plot represents the median value. The whiskers extending from the box represent the range of values excluding outliers. Circles and asterisks indicate outliers (1.5 x the interquartile range) and extreme values (3.0 x the interquartile range). The cut-off values of 50 and 75 nmol/L are indicated.

A change was observed in 25(OH)D concentrations throughout pregnancy, with a predicted peak level in gestational weeks 21–34, followed by a decline towards delivery (Fig 2, Table 2). The level of 25(OH)D was significantly different (p<0.0001) among each consecutive gestational age group, except when comparing weeks 21–28 vs. 29–34 (p = 0.7) and the first vs. second day after delivery (p = 0.9). There was a clear effect of sampling season on 25(OH)D concentrations. Levels were lowest during winter (reference value) and spring (estimated difference 2.4 nmol, standard error (SE) 0.9, p = 0.009), while summer (estimated difference 20.5 nmol/L, SE 1.0, p<0.0001) and autumn (estimated difference 10.6 nmol/L, SE 0.9, p<0.0001) had higher concentrations. Gestational age- and season-specific reference intervals for 25(OH)D, 25(OH)D2, and 25(OH)D3 are shown in Table 3.

**Table 2:** Estimated differences in 25(OH)D in various subgroups of the participants.

Parameter		Estimate (nmol/L) ^1^	SE ^2^	*p*-value ^3^
Intercept		35.2	8.0	<0.001
Gestational week	13–20	Reference		
	21–28	15.7	1.0	<0.001
	29–34	16.3	1.3	<0.001
	35–42	0.6	1.0	0.6
	Delivery	11.7	1.1	<0.001
	1^st^ day after delivery	5.6	1.1	<0.001
	2^nd^ day after delivery	5.8	1.2	<0.001
Sampling season	Winter	Reference		
	Spring	2.4	0.9	0.009
	Summer	20.5	1.0	<0.001
	Autumn	10.6	0.9	<0.001
Possibly vitamin D linked complication	Not present	Reference		
	Present	9.8	2.7	<0.001
Mode of delivery	Vaginal birth	Reference		
	Elective cesarean	10.0	2.1	<0.001
	Emergency cesarean	6.8	2.2	0.002
Mode of conception	Spontaneous	Reference		
	Stimulation	-12.7	16.4	0.4
	In vitro fertilization	0.1	5.4	1.0
	Insemination	-3.7	6.6	0.6
Age	Per unit increase	0.2	0.2	0.4
Parity	0	Reference		
	1	-5.5	1.5	<0.001
	2+	-8.7	2.1	<0.001
Body mass index	Per 1-unit increase	-0.4	0.2	0.03

Smoking	No	Reference		
	Yes	-2.0	1.9	0.3
Social class ^4^	I	Reference		
	II	1.4	1.9	0.5
	III	1.2	2.0	0.5
	IV	3.3	2.3	0.14
	V	-1.3	2.2	0.6

^1^ Estimated difference in 25(OH)D, assessed via a linear mixed model, fitted through the restricted maximum likelihood approach. Tukey’s test for multiple comparisons of means was used.

^2^ Standard error.

^3^ Compared to the reference level of the variable, adjusted for the other variables in the table.

^4^ Respondents were classified into five social groups based on employment grade, job title and education: I) executive managers and/or academics; II) middle managers and/or those with 3–4 years of further education; III) other white-collar workers; IV) skilled blue-collar workers; and V) semi-skilled or unskilled workers.

**Table 3:** Gestational age- and season-specific reference intervals for 25-hydroxyvitamin D (nmol/L) in women with vaginal birth and no possible vitamin D-linked complications in pregnancy.

	Season	13–20 weeks		21–34 weeks		35–42 weeks		Delivery		1^st^ and 2^nd^ day after delivery	
**25-hydroxyvitamin D (D3+D2)**	Winter	n = 81 [0]		n = 197 [1]		n = 84 [0]		n = 116 [0]		n = 179 [0]	
		15.6 (12.1–22.4)	98.0 (80.7–101.1)	21.7 (20.4–23.6)	99.6 (91.8–109.1)	17.5 (13.2–24.9)	81.6 (75.0–85.9)	13.5 (12.1–17.0)	86.2 (77.0–87.4)	13.6 (11.8–15.3)	75.0 (68.0–78.0)
	Spring	n = 47 [0]		n = 131[0]		n = 91 [0]		n = 155 [0]		n = 243 [4]	
		17.4 (16.1–23.7)	94.0 (78.8–95.8)	16.6 (14.1–22.9)	104.5 (83.9–113.9)	18.6 (11.2–20.9)	95.2 (83.6–101.3)	14.6 (14.1–17.2)	101.1 (89.3–108.7)	12.7 (12.1–15.6)	82.5 (76.1–89.2)
	Summer	n = 96 [4]		n = 125 [2]		n = 51 [0]		n = 66 [1]		n = 111 [1]	
		34.9 (27.9–40.3)	98.5 (88.5–102.9)	34.4 (33.0–41.4)	112.2 (104.8–122.1)	27.6 (26.8–35.8)	100.4 (95.5–102.2)	27.9 (26.1–32.1)	112.1 (102.6–115.9)	20.8 (20.2–28.3)	99.2 (91.5–105.9)
	Autumn	n = 186 [5]		n = 231 [3]		n = 38 [0]		n = 56 [0]		n = 102 [1]	
		28.4 (27.4–33.2)	91.2 (85.1–99.1)	24.7 (23.1–27.1)	101.5 (94.1–103.6)	23.1	99.5	22.3 (20.6–25.0)	99.2 (92.5–101.9)	21.7 (19.9–25.0)	86.1 (78.9–108.1)
**25-hydroxyvitamin D3**	Winter	n = 81 [0]		n = 197 [0]		n = 84 [0]		n = 116 [0]		n = 179 [0]	
		13.4 (9.9–20.0)	95.8 (78.2–98.9)	19.5 (18.2–22.2)	97.8 (90.0–107.5)	15.3 (11.0–22.6)	79.4 (73.1–83.7)	11.4 (9.9–14.7)	84.1 (76.0–87.6)	10.6 (9.6–13.1)	73.3 (65.0–76.8)
	Spring	n = 47 [0]		n = 131 [0]		n = 91 [0]		n = 155 [0]		n = 243 [0]	
		14.2 (13.9–21.7)	89.4 (76.4–90.7)	14.1 (11.9–20.2)	102.6 (81.0–112.0)	16.1 (8.1–18.5)	91.5 (82.3–99.1)	12.4 (11.6–14.9)	94.8 (87.2–106.6)	10.4 (8.4–13.2)	80.3 (73.9–87.0)
	Summer	n = 96 [4]		n = 125 [2]		n = 51 [0]		n = 66 [1]		n = 111 [1]	
		33.0 (25.7–38.2)	95.7 (86.1–100.7)	32.2 (30.8–39.2)	110.1 (102.2–119.9)	25.4 (24.6–34.5)	99.1 (93.3–100.0)	24.8 (23.4–29.4)	108.3 (100.4–113.7)	18.7 (18.0–25.9)	96.8 (89.1–103.7)
	Autumn	n = 186 [4]		n = 231 [3]		n = 38 [0]		n = 56 [0]		n = 102 [1]	
		26.3 (24.8–31.0)	92.0 (83.8–97.7)	22.5 (19.8–24.6)	99.1 (90.3–101.4)	20.9	79.1	19.8 (18.4–23.1)	96.9 (87.2–99.1)	19.1 (16.2–22.1)	83.9 (76.0–105.9)
**25-hydroxyvitamin D2**	Winter	n = 81 [-]		n = 197 [-]		n = 84 [-]		n = 116 [24]		n = 179 [–]	
		2.2 (2.2–2.2)	4.8 (3.1–8.4)	2.2 (2.2–2.2)	4.1 (3.7–5.6)	2.2 (2.2–2.2)	5.8 (3.6–6.9)	2.2 (2.2–2.2)	3.9 (3.3–5.3)	2.2 (2.2–2.2)	5.7 (4.0–6.0)
	Spring	n = 47 [-]		n = 131 [-]		n = 91 [-]		n = 155 [32]		n = 243 [–]	
		2.2 (2.2–2.2)	4.8 (2.8–5.1)	2.2 (2.2–2.2)	4.0 (3.3–5.4)	2.2 (2.2–2.2)	5.7 (4.1–6.6)	2.2 (2.2–2.2)	5.9 (4.6–7.3)	2.2 (2.2–2.2)	3.9 (3.0–4.2)
	Summer	n = 96 [-]		n = 125 [-]		n = 51 [8]		n = 66 [-]		n = 111 [–]	
		2.2 (2.2–2.2)	3.8 (2.8–6.2)	2.2 (2.2–2.2)	3.4 (2.8–5.3)	2.2 (2.2–2.2)	5.2 (3.6–5.7)	2.2 (2.2–2.2)	3.7 (3.2–4.4)	2.2 (2.2–2.2)	3.4 (3.0–4.5)
	Autumn	n = 186 [-]		n = 231 [-]		n = 38 [-]		n = 56 [-]		n = 102 [–]	
		2.2 (2.2–2.2)	4.1 (2.8–4.2)	2.2 (2.2–2.2)	4.5 (3.3–6.1)	2.2	2.2	2.2 (2.2–2.2)	5.3 (4.1–5.4)	2.2 (2.2–2.2)	6.8 (3.7–9.1)

Gestational age and seasonal reference intervals were determined from 2403 blood samples from 524 healthy, pregnant Caucasian women at the 2.5^th^ and 97.5^th^ percentiles with a 90% confidence interval (in parentheses below). In situations with fewer than 40 cases, only a descriptive 95 inter-percentile range is shown. The number of observations is listed with the number of outliers removed (square brackets, “-”if possible outliers cannot be detected). Seasons were defined as winter (December-February), spring (March-May), summer (June-August), and autumn (September-November). The reference intervals were calculated using a robust non-parametric bootstrap method with 500 iterations, according to the International Federation of Clinical Chemistry guidelines. The level of 25-hydroxyvitamin D was significantly different (p<0.0001) among each consecutive gestational age group, except when comparing weeks 21–28 vs. 29–34 (p = 0.7) and first vs. second day after delivery (p = 0.9), which were merged.

After adjusting for other variables, there was a significant estimated difference in 25(OH)D concentrations between women with and without possible vitamin D-linked complications (9.8 nmol/L, SE 2.7, p<0.001), such that those with complications had the highest values. When compared with women giving birth vaginally, those with elective (10.0 nmol/L, SE 2.1, p<0.001) and emergency cesarean section (6.8 nmol/L, SE 2.2, p<0.001) had higher 25(OH)D concentrations. A small decrease in 25(OH)D level (0.4 nmol/L, SE 0.2, p = 0.03) was observed for every increase in BMI unit. Nulliparous women had higher 25(OH)D concentrations than primiparous (-5.5 nmol/L, SE 1.5, p<0.001) and multiparous (-8.7 nmol/L, SE 2.1, p<0.001) women. Mode of conception, age, smoking status, and social class were not associated with 25(OH)D concentrations (Table 2).

## Discussion

We present the 2.5^th^ and 97.5^th^ percentiles of gestational age- and season-specific reference intervals with significant variations of 25(OH)D in plasma. The levels of 25(OH)D2 were generally low, without significant change during pregnancy or with changing seasons. Only approximately half of the samples in our cohort had a 25(OH)D level of 50 nmol/L or above, which is lower than the level recommended by many guidelines [16–18]. The deficiency rate was more pronounced during winter and spring, where only 9% had a 25(OH)D level above 75 nmol/L, a level considered optimal during pregnancy in Denmark [17]. In the UK, Moon et al. introduced the concept of “season-corrected” 25(OH)D in pregnant women [19]. The definition of vitamin D deficiency has changed over time, and levels depend on ethnicity and exposure to sunlight [1, 10, 20]. The average concentration of 25(OH)D in pregnant European women ranges from 15–72 nmol/L [10]. At the time of blood sampling for this study few products were fortified (margarine and spreads), and vitamin D fortification has still not yet been widely implemented in Denmark. Recommendations regarding daily supplementation during pregnancy vary from zero to 50 μg [2, 16, 18, 21]. Our samples were collected before the official Danish recommendation of 10 μg of vitamin D3 daily [21]. At that time, two-thirds of women reported using supplements, although two-thirds took less than 10 μg/day [22]. The present results indicate, in accordance with those of other studies, that 10 μg of vitamin D3 daily is not sufficient to maintain 25(OH)D concentrations above 50 nmol/L in pregnancy, especially during winter and spring [23, 24]. Some of the variations in recommendations might be due to differences in analytical methods. The predominantly used immunological method does not differentiate between 25(OH)D2 and 25(OH)D3 and yields rather large variations in the results [13]. The general level of 25(OH)D is most likely typical for pregnant Caucasian women at northern latitudes. Similar to our study, a study from northern Sweden (latitude 64° N) found concentrations below 50 nmol/L in at least one-third of pregnant women [8]. However, study enrolment took place solely from September to March. Other Danish papers report 25(OH)D concentrations similar to those of the present study, including a birth cohort study from 1988–89 on samples collected at gestational week 25 [25] and a more recent cross-sectional study in early pregnancy [7]. A Norwegian longitudinal study (63° N and 58° N) on pregnant women in the second and third trimesters showed slightly higher 25(OH)D concentrations than we observed; however, they measured 25(OH)D with an immunological method [26]. Interestingly, a longitudinal study from the same geographic area as ours assessed 25(OH)D by LC-MS/MS in pregnancy more than ten years before we did [27] and reported that only 1.4–4.3% and 16–19% of women had 25(OH)D concentrations below 25 nmol/L and 50 nmol/L, respectively. Their findings are consistent with our summer values, and one may therefore suspect imbalanced seasonal data collection.

Women with possible vitamin D-linked complications during pregnancy and women with cesarean section had higher estimated 25(OH)D concentrations than women without complications and vaginal birth. Our data do not support the assumption that low 25(OH)D is correlated with an increased risk of pregnancy complications or cesarean section, but these conclusions are uncertain due to the heterogeneity of complications and low numbers. Only composite analyzes were performed due to these low numbers in the subgroups. The observed significant differences might not be clinically relevant, due to the small absolute numbers and analytic uncertainty.

Vitamin D deficiency has been associated with several diseases and conditions, but some of these associations are now being questioned [1, 3, 6, 28]. Associations found in observational studies have predominantly not been reproduced in intervention studies [4]. Clinical vitamin D deficiency in non-pregnant persons can occur at levels lower than 25 nmol/L, while rickets usually first develops below 15 nmol/L. Randomized controlled trials and systematic reviews of supplementation during pregnancy have produced conflicting results and conclusions. A systematic review of 39 studies (seven randomized control trials) suggested that vitamin D levels are inversely correlated with preeclampsia, gestational diabetes, preterm delivery, primary cesarean section, and infertility [29]. A more recent systematic review of 43 randomized studies concluded that prenatal supplementation was associated with increased 25(OH)D concentrations, increased mean birth weight, reduced risk of wheezing in offspring, and increased infant length. There was no benefit on maternal health conditions related to pregnancy; however, most of the studies were small and of poor quality and were therefore insufficient to provide clear recommendations [2]. A large randomized controlled trial assessing the effects of vitamin D supplementation from mid-pregnancy to birth showed that supplementation had dose-dependent effects on vitamin D concentration but did not improve growth or have any effect on maternal pregnancy complications [30]. However, supplementation from pre-conception or early gestation might have yielded a different result. More investigation is needed to understand the differences in vitamin D metabolism between pregnant and non-pregnant women [31]. However, based on one study, pre-conceptional and early gestational 25(OH)D seems not to affect fertility or pregnancy outcomes [32]. Genetic variants in vitamin D metabolism have been proposed to influence risk of developing gestational diabetes, rather than vitamin D concentrations [33]. Genetic variations in vitamin D binding protein may explain some of the variance in 25(OH)D, as seen in a recent study from Copenhagen [34]. A Mendelian randomization study revealed no causal effect of vitamin D status on preeclampsia or gestational hypertension [35]. A review on the long-term effects of low maternal 25(OH)D levels on extra-skeletal health in offspring found some associations with disease, although evidence was conflicting and sparse [36]. However, a recent randomized trial showed effect of high dose vitamin D supplementation in the third trimester judged by improved bone mineralization in offspring up age six [37].

### Strengths and limitations

Our study is a large, longitudinal cohort consisting of healthy Caucasian women. Sampling was conducted evenly throughout the seasons. We have data on pregnancy outcomes, and 25(OH)D was measured using the gold standard method. The limitations include the lack of information regarding pre-conceptional and early gestational 25(OH)D levels, diet, supplementation, sun exposure, and long-term health of the children. Our study design did not allow us to evaluate early vitamin D status and its importance for fetal development.

## Conclusion

The 25(OH)D concentrations vary significantly with both season and gestational age. Healthy women had lower 25(OH)D levels than recommended, without an association with an increased risk of pregnancy complications. These low levels seem sufficient for healthy Caucasian Danish pregnant women. The Danish guideline for vitamin D in pregnancy might need revision. The long-term health of children in relation to gestational vitamin D status needs to be investigated.

## Supporting information

S1 File Minimal anonymized data set. (XLSX)
